# Risk of second primary cancers in patients with rectal neuroendocrine neoplasms: a surveillance, epidemiology, and end results analysis

**DOI:** 10.3389/fonc.2023.1248268

**Published:** 2023-09-13

**Authors:** Ming Wan, Jiaqi Wu, Zhaopeng Jiang, Wushuang Gong, Xianli Zhou

**Affiliations:** In-Patient Ultrasound Department, Second Affiliated Hospital of Harbin Medical University, Surgeons’ Hall, Harbin, Heilongjiang, China

**Keywords:** rectal NENs, second primary cancer, Sir, Ear, SEER

## Abstract

**Background:**

While an elevated risk of second primary cancers (SPCs) has been observed in many other cancers, risk of SPCs has not been quantified in patients with rectal neuroendocrine neoplasms (NENs).

**Methods:**

Survivors of primary rectal NENs diagnosed between 2000 and 2018 were identified from the Surveillance, Epidemiology, and End Results (SEER)-18 registries. Relative risk of SPCs was estimated as the standardized incidence ratio (SIR), which was calculated using SEER*Stat software.

**Results:**

Between 2000 and 2018, a total of 15836 patients diagnosed with rectal NENs, of whom 1436 (9.1%) received diagnosis of SPCs (SIR: 1.19, 95%CI: 1.13-1.26). The majority of patients were aged 50-69 and had their first cancer diagnosed at the localized stage. Male survivors had a higher propensity for developing SPCs overall, while female survivors exhibited higher risks of specific SPCs. Age at diagnosis of rectal NENs influenced the risk of SPCs, with younger patients having greater risks. A statistically significant increase in the incidence of SPCs was observed among patients aged 30-64 years. Black patients had higher relative risks of certain SPCs, while White patients had a lower risk of subsequent melanoma. Trend analysis revealed that the highest excess burden of SPCs was observed in the years 2000 to 2002. Risk of SPCs remained elevated within the first four years post-diagnosis for survivors of rectal NENs, but diminished thereafter.

**Conclusion:**

The study revealed that individuals who survived rectal NENs were at an elevated risk of developing SPCs compared to the general population. Our results hold important implications for the formulation of lifelong surveillance recommendations for cancer survivors.

## Introduction

Neuroendocrine neoplasms (NENs) are a heterogeneous group of uncommon diseases with varied clinical characteristics and biological behaviors which arising from neuroendocrine cells throughout the diffuse endocrine system (1, 2). Due to advances in diagnostic technologies, there has been a significant increase in the detection of NENs both in the United States and globally (3–5). Rectal NENs are one of the main subtypes of NENs in the gastrointestinal tract (6, 7). Despite the typically indolent nature of rectal NENs and the improving outcomes in cancer management, survivors of these tumors still remain at a higher risk of developing second primary cancers (SPCs) during their cancer survivorship (8, 9). The heightened risk of SPCs may be partially attributed to genetic susceptibility and shared risk factors between NENs and secondary malignancies. Furthermore, regular medical surveillance for cancer survivors often leads to the frequent detection of SPCs.

In order to mitigate the relative risk of SPCs, it is imperative for individuals who have been diagnosed with rectal NENs to undergo regular follow-up visits and screenings as recommended by their healthcare providers. While previous studies have assessed the relative risk of SPCs in patients with various other cancers, there is a lack of research specifically examining the risk of developing a secondary malignancy among rectal NENs survivors in comparison to the general population in the United States. Therefore, in this present study, we attempted to comprehensively evaluate the relative risk of SPCs among patients with a history of rectal NENs using data from the SEER-18 program and to demonstrate the need for the development of appropriate surveillance protocols for this high-risk patient population.

## Materials and methods

Patients who were histologically diagnosed with rectal NENs were extracted from the Surveillance, Epidemiology, and End Results (SEER)-18 registries, which includes nearly 28% of the US population from 2000 to 2018. This database includes incidence and population data stratified by race, sex, year of diagnosis, geographic area, and age. An at least 2-month latency between first rectal NENs diagnosis and SPCs was used to exclude synchronous primary malignancies. The study protocol was approved by the Institutional Review Board (IRB) of Second Affiliated Hospital of Harbin Medical University, and the requirement for written informed consent was waived due to the study’s design.

### Statistical analysis

Standardized incidence ratios (SIRs) and absolute excess rate (AER) per 10,000 person-years were calculated to estimate the relative risk of a SPCs among rectal NENs survivors relative to the year 2000 US general population. Poisson regression models were used to calculated the corresponding 95% confidence intervals (CIs). All analyses were conducted by the SEER*Stat software.

## Results

Between 2000 and 2018, we identified 15836 patients diagnosed with rectal NENs, of whom 1436 (9.1%) received diagnosis of a second primary cancer. The detailed demographics and characteristics of the entire study population are presented in **Table 1**. The majority of patients (66.7%) were between the ages of 50 and 69, and 78.3% had their first cancer diagnosed at the localized stage. More than half of the patients were of white ethnicity (55.6%) and were married (52.4%). The majority of patients (81.7%) underwent surgical intervention as the primary treatment for their initial malignancy, while only a small minority (2.9%) received chemotherapy. Male patients demonstrated a higher propensity for developing subsequent malignancies compared to female patients. Likewise, among those who received a diagnosis of a SPC, the majority had their initial malignancy identified at the localized stage.

**Table 1:** Patient demographics of the study cohort, SEER 2000–2018.

Characteristics	Frequency (%)		
	All patients (n=15863)	Rectal NENs (n=14427)	SPCs (n=1436)
Age at diagnosis of first malignancy			
< 50	3708 (23.4)	3529 (24.5)	179 (12.5)
50-69	10573 (66.7)	9553 (66.2)	1020 (71.0)
≥ 70	1582 (9.9)	1345 (9.3)	237 (16.5)
Gender			
Female	7943 (50.1)	7333 (50.8)	610 (42.5)
Male	7920 (49.9)	7094 (49.2)	826 (57.5)
Race			
White	8822 (55.6)	8027 (55.6)	795 (55.4)
Black	3796 (23.9)	3367 (23.3)	429 (29.9)
Other	3245 (20.5)	3033 (21.1)	212 (14.7)
Marital status at diagnosis of first malignancy			
Married	8315 (52.4)	7527 (52.2)	788 (54.9)
Unmarried	7548 (47.6)	6900 (47.8)	648 (45.1)
Year of diagnosis of first malignancy			
2000-2009	6996 (44.1)	6056 (29.2)	970 (67.5)
2010-2018	8867 (55.9)	8401 (70.8)	466 (32.5)
Tumor grade at diagnosis of first malignancy			
Well differentiated	5633 (35.5)	5297 (36.7)	336 (23.4)
Poorly differentiated	381 (2.4)	363 (2.5)	18 (1.3)
Unknown	9849 (62.1)	8767 (60.8)	1082 (75.3)
Stage at diagnosis of first malignancy			
Localized	12424 (78.3)	11212 (77.7)	1212 (84.4)
Regional	298 (1.9)	277 (1.9)	21 (1.5)
Distant	493 (3.1)	484 (3.4)	9 (0.6)
Unstaged	2648 (16.7)	2454 (17.0)	194 (13.5)
Received surgery for first malignancy			
Yes	12953 (81.7)	11781 (81.7)	1172 (81.6)
No	2910 (18.3)	2646 (18.3)	264 (18.4)
Received chemotherapy for first malignancy			
Yes	466 (2.9)	450 (3.1)	16 (1.1)
No	15397 (97.1)	13977 (96.9)	1420 (98.9)

NENs, neuroendocrine neoplasms; SPC, second primary cancers.

### Risk and burden of SPC

**Figure 1:**
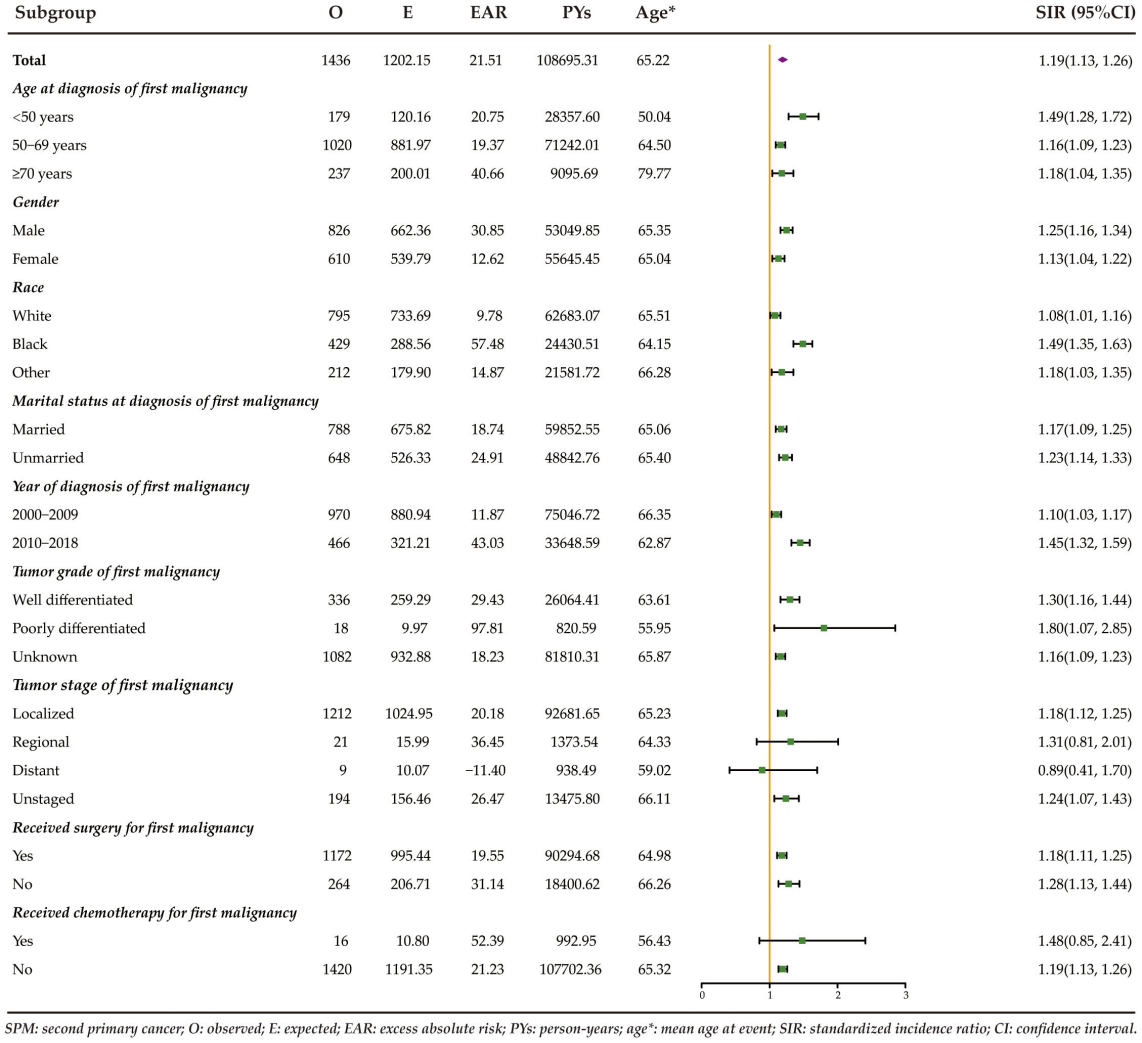
Risk of developing SPCs stratified by patient characteristics of rectal NENs in the United States between 2000 and 2018.

**Figure 2:**
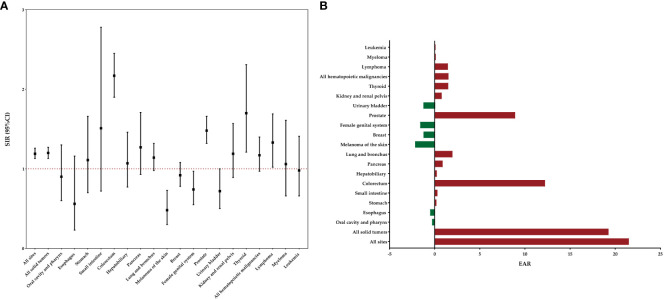
Risk of developing specific SPCs after rectal NENs in the overall study population. **(A)** SIRs with corresponding 95% CIs. **(B)** EAR per 10,000 person-years.

**Figure 1** displays the risk and burden of SPCs overall and by patient characteristics. Among the study population, a total of 1436 patients developed a SPC, surpassing the expected cases if these patients had the same cancer risk as the general population. The SIR of developing a SPC was 1.19 (95%CI: 1.13-1.26) and the EAR was 21.51 cases per 10000 person-years. Among all patients with rectal NENs in the SEER database, there was a notable increase in the risk of developing four specific subsequent primary cancers: colorectum, prostate, thyroid, and lymphoma (**Figure 2**). The relative risk was highest for the occurrence of second colorectal cancers (SIR: 2.34, 95%CI: 2.08-2.63). Additionally, patients with rectal NENs exhibited significantly lower incidence rates of certain cancers compared to the general US population, including melanoma of the skin and female genital system cancers. Rectal NENs survivors who had their disease diagnosed at the unknown stage or at the localized stage exhibited a higher risk of developing SPCs, with SIRs of 1.24 (95%CI: 1.07-1.43) and 1.18 (95%CI: 1.12-1.25), respectively. Furthermore, patients who underwent surgery or chemotherapy for their initial malignancy had a more pronounced relative risk of developing subsequent cancers compared to those who did not receive these treatments.

For male survivors, a 25% increased relative risk was observed for SPCs overall (**Table 2**). Similarly, female survivors exhibited a 13% increased relative risk for SPCs overall. Further analysis by sex revealed that female patients had higher relative risks of developing SPCs in the colorectum, kidney and renal pelvis, and thyroid. However, male survivors only displayed an elevated risk of second primary colorectal and prostate cancers compared to the general population. Both of them showed a reduced risk of developing second melanoma of the skin relative to the age-matched US population.

**Table 2:** Relative risk of selected SPCs after first rectal NENs by sex in US from 2000 through 2018.

SPC	Males			Females		
	Observed	Expected	SIR(95%CI)	Observed	Expected	SIR(95%CI)
All sites	826	662.36	1.25* (1.16, 1.34)	610	539.79	1.13* (1.04, 1.22)
All solid tumors	732	580.54	1.26* (1.17, 1.36)	537	478.96	1.12* (1.03, 1.22)
Oral cavity and pharynx	24	23.04	1.04 (0.67, 1.55)	4	8.11	0.49 (0.13, 1.26)
Esophagus	6	9.83	0.61 (0.22, 1.33)	1	2.60	0.38 (0.01, 2.14)
Stomach	11	13.18	0.83 (0.42, 1.49)	12	7.60	1.58 (0.82, 2.76)
Small intestine	7	3.58	1.95 (0.79, 4.02)	3	3.04	0.99 (0.20, 2.88)
Colorectum	119	62.18	1.91* (1.59, 2.29)	128	51.89	2.47* (2.06, 2.93)
Hepatobiliary	30	25.92	1.16 (0.78, 1.65)	10	11.43	0.88 (0.42, 1.61)
Pancreas	28	18.97	1.48 (0.98, 2.13)	17	16.33	1.04 (0.61, 1.67)
Lung and bronchus	106	87.42	1.21 (0.99, 1.47)	73	70.14	1.04 (0.82, 1.31)
Melanoma of the skin	15	29.41	0.50* (0.28, 0.83)	7	15.85	0.44* (0.18, 0.91)
Breast	NA	NA	NA	153	166.86	0.92 (0.78, 1.07)
Female genital system	NA	NA	NA	49	66.51	0.74 (0.55, 0.97)
Prostate	298	201.06	1.48* (1.32, 1.66)	NA	NA	NA
Urinary bladder	25	37.85	0.66 (0.43, 0.98)	10	10.77	0.93 (0.45, 1.71)
Kidney and renal pelvis	28	28.51	0.98 (0.65, 1.42)	24	15.01	1.60* (1.02, 2.38)
Thyroid	10	6.99	1.43 (0.69, 2.63)	30	16.56	1.81* (1.22, 2.59)
All hematopoietic malignancies	67	57.55	1.16 (0.90, 1.48)	49	41.72	1.17 (0.87, 1.55)
Lymphoma	38	28.15	1.35 (0.96, 1.85)	27	20.91	1.29 (0.85, 1.88)
Myeloma	11	11.50	0.96 (0.48, 1.71)	11	9.24	1.19 (0.59, 2.13)
Leukemia	18	17.90	1.01 (0.60, 1.59)	11	11.58	0.95 (0.47, 1.70)

SPCs, second primary cancers; NENs, neuroendocrine neoplasms; SIR, standardized incidence ratio; CI, confidence interval; NA, not applicable.* means having statistical significance.

The impact of age at diagnosis of first primary rectal NENs on the risk of SPCs was examined in our study (**Table 3**). Rectal NENs patients diagnosed at less than 50 years old had a significantly elevated risk of developing SPCs compared to the general population (SIR: 1.49, 95%CI: 1.28-1.72). Similarly, patients aged 50-69 and ≥70 years exhibited overall relative risks that were 16% and 18% higher, respectively. When examining specific SPCs, significantly increased relative risks were observed for colorectal cancer and pancreatic cancer among survivors younger than 50 years old at the time of their first cancer diagnosis. For individuals aged 50-69 years, the risks of developing second colorectal cancer, prostate cancer, and second thyroid cancer were significantly higher compared to the general population. Patients older than 70 years were associated with a higher risk of developing second cancers of the kidney and renal pelvis, as well as lymphoma.

**Table 3:** Relative risk of selected SPCs after first erctal NENs by age at diagnosis in US from 2000 through 2018.

SPCs	< 50 years			50-69 years			≥ 70 years		
	Observed	Expected	SIR(95%CI)	Observed	Expected	SIR(95%CI)	Observed	Expected	SIR(95%CI)
All sites	179	120.16	1.49* (1.28, 1.72)	1020	881.97	1.16* (1.09, 1.23)	237	200.01	1.18* (1.04, 1.35)
All solid tumors	160	107.28	1.49* (1.27, 1.74)	915	783.51	1.17* (1.09, 1.25)	194	168.72	1.15 (0.99, 1.32)
Oral cavity and pharynx	4	3.88	1.03 (0.28, 2.64)	21	23.57	0.89 (0.55, 1.36)	3	3.71	0.81 (0.17, 2.37)
Esophagus	0	0.97	0.00 (0.00, 3.80)	6	9.37	0.64 (0.24, 1.39)	1	2.09	0.48 (0.01, 2.66)
Stomach	1	1.79	0.56 (0.01, 3.11)	18	14.44	1.25 (0.74, 1.97)	4	4.54	0.88 (0.24, 2.26)
Small intestine	1	0.70	1.43 (0.04, 7.99)	8	4.90	1.63 (0.70, 3.22)	1	1.03	0.97 (0.02, 5.41)
Coloretum	55	11.93	4.61* (3.47, 6.00)	160	79.90	2.00* (1.70, 2.34)	32	22.23	1.44 (0.98, 2.03)
Hepatobiliary	7	3.24	2.16 (0.87, 4.45)	26	28.52	0.91 (0.60, 1.34)	7	5.58	1.25 (0.50, 2.59)
Pancreas	7	2.46	2.85* (1.15, 5.87)	27	25.13	1.07 (0.71, 1.56)	11	7.70	1.43 (0.71, 2.55)
Lung and bronchus	11	9.34	1.18 (0.59, 2.11)	135	115.01	1.17 (0.98, 1.39)	33	33.20	0.99 (0.68, 1.40)
Melanoma of the skin	6	5.92	1.01 (0.37, 2.21)	14	32.36	0.43* (0.24, 0.73)	2	7.28	0.27* (0.03, 0.99)
Breast	22	25.28	0.87 (0.55, 1.32)	107	121.82	0.88 (0.72, 1.06)	26	21.30	1.22 (0.80, 1.79)
Female genital system	3	9.18	0.33* (0.07, 0.95)	34	49.37	0.69* (0.48, 0.96)	12	7.95	1.51 (0.78, 2.64)
Prostate	17	13.22	1.29 (0.75, 2.06)	247	163.19	1.51* (1.33, 1.71)	34	24.65	1.38 (0.96, 1.93)
Urinary bladder	4	2.58	1.55 (0.42, 3.97)	25	33.51	0.75 (0.48, 1.10)	3	12.52	0.48 (0.18, 1.04)
Kidney and renal pelvis	8	4.91	1.63 (0.70, 3.21)	32	32.71	0.98 (0.67, 1.38)	12	5.90	2.03* (1.05, 3.55)
Thyroid	11	5.69	1.93 (0.97, 3.46)	29	16.30	1.78* (1.19, 2.56)	0	1.57	0.00 (0.00, 2.35)
All hematopoietic malignancies	11	9.84	1.12 (0.56, 2.20)	77	70.09	1.10 (0.87, 1.37)	28	19.34	1.45 (0.96, 2.09)
Lymphoma	7	5.44	1.29(0.52, 2.65)	41	34.47	1.19 (0.85, 1.61)	17	9.14	1.86* (1.08, 2.98)
Myeloma	1	1.67	0.60(0.02, 3.33)	15	15.14	0.99 (0.55, 1.63)	6	3.92	1.53 (0.56, 3.33)
Leukemia	3	2.72	1.10(0.23, 3.22)	21	20.47	1.03 (0.64, 1.57)	5	6.29	0.80 (0.26, 1.86)

SPCs, second primary cancers; NENs, neuroendocrine neoplasms; SIR, standardized incidence ratio; CI, confidence interval.* means having statistical significance.

Race-specific analyses reveal that Black patients had a significantly higher relative risk of developing secondary cancers of colorectum, lung and bronchus, prostate, and thyroid compared to White patients (**Table 4**). It is noteworthy that White patients were found to have a significantly lower risk of subsequent melanoma of the skin (SIR: 0.51, 95%CI: 0.32-0.78).

**Table 4:** Relative risk of selected SPCs after first erctal NENs by race in US from 2000 through 2018.

SPCs	White			Black			Other		
	Observed	Expected	SIR(95%CI)	Observed	Expected	SIR(95%CI)	Observed	Expected	SIR(95%CI)
**All sites**	795	733.69	1.08* (1.01, 1.16)	429	288.56	1.49* (1.35, 1.63)	212	179.90	1.18* (1.03, 1.35)
All solid tumors	695	643.36	1.08* (1.01, 1.16)	383	256.95	1.49* (1.34, 1.65)	191	159.09	1.20* (1.04, 1.38)
Oral cavity and pharynx	19	20.41	0.93 (0.56, 1.45)	3	5.73	0.52 (0.11, 1.53)	6	5.01	1.20 (0.44, 2.61)
Esophagus	6	7.94	0.76 (0.28, 1.65)	1	2.79	0.36 (0.01, 2.00)	0	1.71	0.00 (0.00, 2.16)
Stomach	11	10.07	1.09 (0.55, 1.96)	7	5.76	1.21 (0.49, 2.50)	5	4.95	1.01 (0.33, 2.36)
Small intestine	5	3.52	1.42 (0.46, 3.31)	3	2.32	1.29 (0.27, 3.78)	2	0.79	2.54 (0.31, 9.18)
Colorectum	125	64.76	1.93* (1.61, 2.30)	78	30.23	2.58* (2.04, 3.22)	44	19.07	2.31* (1.68, 3.10)
Hepatobiliary	18	18.80	0.96 (0.57, 1.51)	12	9.34	1.28 (0.66, 2.24)	10	9.20	1.09 (0.52, 2.00)
Pancreas	24	20.15	1.19 (0.76, 1.77)	12	9.58	1.25 (0.65, 2.19)	9	5.56	1.62 (0.74, 3.07)
Lung and bronchus	93	95.10	0.98 (0.79, 1.20)	60	39.87	1.50* (1.15, 1.94)	26	22.58	1.15 (0.75, 1.69)
Melanoma of the skin	21	40.96	0.51* (0.32, 0.78)	0	0.52	0.00 (0.00, 7.13)	1	4.08	0.25 (0.01, 1.37)
Breast	84	98.30	0.85 (0.68, 1.06)	51	45.04	1.13 (0.84, 1.49)	20	25.07	0.80 (0.49, 1.23)
Female genital system	30	38.88	0.77 (0.52, 1.10)	13	17.74	0.73 (0.39, 1.25)	6	9.89	0.61 (0.22, 1.32)
Prostate	158	117.26	1.35* (1.15, 1.57)	102	56.81	1.80* (1.46, 2.18)	38	26.98	1.41* (1.01, 1.93)
Urinary bladder	23	35.51	0.65 (0.41, 0.98)	8	6.71	1.19 (0.51, 2.35)	4	6.59	0.61 (0.17, 1.55)
Kidney and renal pelvis	30	25.02	1.20 (0.81, 1.71)	14	10.89	1.29 (0.70, 2.16)	8	6.14	1.30 (0.56, 2.57)
Thyroid	24	14.83	1.62* (1.04, 2.41)	9	4.12	2.18* (1.01, 4.15)	7	4.61	1.52 (0.61, 3.13)
All hematopoietic malignancies	70	62.68	1.12 (0.87, 1.41)	32	22.13	1.45 (0.99, 2.04)	14	14.47	0.97 (0.53, 1.62)
Lymphoma	40	32.71	1.22(0.87, 1.66)	13	8.41	1.55 (0.82, 2.64)	12	7.93	1.51 (0.78, 2.64)
Myeloma	10	10.01	1.00(0.48, 1.84)	11	8.24	1.34 (0.67, 2.39)	1	2.49	0.40 (0.01, 2.24)
Leukemia	20	19.95	1.00(0.61, 1.55)	8	5.48	1.46 (0.63, 2.88)	1	4.05	0.25 (0.01, 1.37)

SPCs, second primary cancers; NENs, neuroendocrine neoplasms; SIR, standardized incidence ratio; CI, confidence interval.* means having statistical significance.

Patients diagnosed with poorly differentiated rectal NENs exhibited a statistically significant increase in the SIR for developing SPCs at all sites (SIR: 1.80, 95% CI: 1.07, 2.85) (**Table 5**). Among patients with well-differentiated tumors, prostate cancer was the most common subsequent primary cancer, showing a 74% increase compared to the general population.

**Table 5:** Relative risk of selected SPCs after first rectal NENs by tumor differentiation in US from 2000 through 2018.

SPCs	Well differentiated			Poorly differentiated		
	Observed	Expected	SIR(95%CI)	Observed	Expected	SIR(95%CI)
All sites	336	259.29	1.30* (1.16, 1.44)	18	9.97	1.80* (1.07, 2.85)
All solid tumors	298	229.12	1.30* (1.16, 1.46)	16	8.71	1.84* (1.05, 2.98)
Oral cavity and pharynx	4	7.16	0.56 (0.15, 1.43)	1	0.24	4.08 (0.10, 22.76)
Esophagus	1	2.63	0.38 (0.01, 2.12)	0	0.10	0.00 (0.00, 36.16)
Stomach	6	4.37	1.37 (0.50, 2.99)	1	0.16	6.30 (0.16, 35.10)
Small intestine	2	1.49	1.34 (0.16, 4.85)	0	0.05	0.00 (0.00, 72.13)
Colorectal	55	24.25	2.27* (1.71, 2.95)	7	0.95	7.40* (2.97, 15.24)
Hepatobiliary	9	8.54	1.05 (0.48, 2.00)	1	0.26	3.85 (0.10, 21.42)
Pancreas	9	7.55	1.19 (0.55, 2.26)	0	0.30	0.00 (0.00, 12.20)
Lung and bronchus	38	32.08	1.18 (0.84, 1.63)	2	1.34	1.50 (0.18, 5.41)
Melanoma of the skin	6	10.28	0.58 (0.21, 1.27)	0	0.45	0.00 (0.00, 8.16)
Breast	39	37.96	1.03 (0.73, 1.40)	2	1.45	1.38 (0.17, 4.99)
Female genital system	11	15.04	0.73 (0.37, 1.31)	0	0.56	0.00 (0.00, 6.54)
Prostate	73	42.00	1.74* (1.36, 2.19)	0	1.46	0.00 (0.00, 2.53)
Urinary bladder	7	9.78	0.72 (0.29, 1.48)	1	0.47	2.15 (0.05, 11.97)
Kidney and renal pelvis	14	9.86	1.42 (0.78, 2.38)	1	0.34	2.91 (0.07, 16.22)
Thyroid	11	5.81	1.89 (0.94, 3.39)	0	0.19	0.00 (0.00, 19.61)
All hematopoietic malignancies	27	21.42	1.26 (0.83, 1.83)	1	0.86	1.17 (0.03, 6.50)
Lymphoma	17	10.63	1.60 (0.93, 2.56)	0	0.43	0.00 (0.00, 8.61)
Myeloma	3	4.48	0.67 (0.14, 1.96)	0	0.16	0.00 (0.00, 22.92)
Leukemia	7	6.31	1.11 (0.45, 2.29)	2	0.44	4.50 (0.55, 16.27)

SPCs, second primary cancers; NENs, neuroendocrine neoplasms; SIR ,standardized incidence ratio; CI, confidence interval.* means having statistical significance.

Stage-specific risk analyses indicate that patients with localized rectal NENs had a higher likelihood of developing SPCs, with a SIR of 1.18 between 2000 and 2018. Notably, patients with regional disease had a significantly elevated risk of developing second hepatobiliary cancers compared to the general US population (SIR: 5.91, 95% CI: 1.22-17.27), whereas this increased risk was not observed for patients with distant rectal NENs (**Table 6**).

**Table 6:** Relative risk of selected SPCs after first rectal NENs by tumor stage in US from 2000 through 2018.

SPCs	Localized			Regional			Distant		
	Observed	Expected	SIR(95%CI)	Observed	Expected	SIR(95%CI)	Observed	Expected	SIR(95%CI)
**All sites**	1212	1024.95	1.18* (1.12, 1.25)	21	15.99	1.31 (0.81, 2.01)	9	10.07	0.89 (0.41, 1.70)
All solid tumors	1070	903.31	1.18* (1.11, 1.26)	19	14.02	1.36 (0.82, 2.12)	8	8.85	0.90 (0.39, 1.78)
Oral cavity and pharynx	21	26.64	0.79 (0.49, 1.21)	1	0.39	2.56 (0.06, 14.27)	0	0.26	0.00 (0.00, 14.16)
Esophagus	6	10.62	0.57 (0.21, 1.23)	0	0.16	0.00 (0.00, 22.98)	0	0.11	0.00 (0.00, 34.68)
Stomach	21	17.73	1.18 (0.73, 1.81)	1	0.30	3.34 (0.08, 18.63)	0	0.19	0.00 (0.00, 19.64)
Small intestine	9	5.62	1.60 (0.73, 3.04)	0	0.08	0.00 (0.00, 45.08)	1	0.06	18.03 (0.46, 100.46)
Colorectum	204	97.13	2.10* (1.82, 2.41)	3	1.54	1.94 (0.40, 5.68)	4	1.02	3.91* (1.07, 10.01)
Hepatobiliary	34	31.99	1.06 (0.74, 1.49)	3	0.51	5.91* (1.22, 17.27)	0	0.32	0.00 (0.00, 11.52)
Pancreas	41	30.04	1.36 (0.98, 1.85)	1	0.48	2.07 (0.05, 11.51)	0	0.30	0.00 (0.00, 12.12)
Lung and bronchus	147	134.20	1.10 (0.93, 1.29)	5	2.18	2.30 (0.75, 5.36)	0	1.35	0.00 (0.00, 2.74)
Melanoma of the skin	20	39.03	0.51 (0.31, 0.79)	0	0.59	0.00 (0.00, 6.30)	0	0.35	0.00 (0.00, 10.59)
Breast	133	142.88	0.93 (0.78, 1.10)	1	2.27	0.44 (0.01, 2.45)	1	1.26	0.00 (0.20, 4.41)
Female genital system	44	56.43	0.78 (0.57, 1.05)	0	0.89	0.00 (0.00, 4.14)	0	0.49	0.00 (0.00, 7.60)
Prostate	253	172.00	1.47* (1.30, 1.66)	1	2.45	0.41 (0.01, 2.27)	1	1.78	0.56 (0.01, 3.12)
Urinary bladder	33	41.68	0.79 (0.54, 1.11)	1	0.71	1.41 (0.04, 7.85)	0	0.42	0.00 (0.00, 8.88)
Kidney and renal pelvis	40	37.11	1.08 (0.76, 1.48)	1	0.55	1.82 (0.05, 10.13)	1	0.36	2.75 (0.07, 15.30)
Thyroid	30	20.07	1.49* (1.01, 2.13)	1	0.30	3.28 (0.08, 18.30)	0	0.18	0.00 (0.00, 20.41)
All hematopoietic malignancies	100	84.66	1.18 (0.96, 1.44)	1	1.35	0.74 (0.02, 4.14)	0	0.84	0.00 (0.00, 4.41)
Lymphoma	57	41.92	1.36* (1.03, 1.76)	1	0.68	1.47 (0.04, 8.19)	0	0.41	0.00 (0.00, 8.98)
Myeloma	18	17.57	1.02 (0.61, 1.62)	0	0.26	0.00 (0.00, 14.10)	0	0.18	0.00 (0.00, 14.87)
Leukemia	25	25.17	0.99 (0.64, 1.47)	0	0.40	0.00 (0.00, 9.13)	0	0.25	0.00 (0.00, 14.87)

SPCs, second primary cancers; NENs, neuroendocrine neoplasms; SIR, standardized incidence ratio; CI, confidence interval.* means having statistical significance.

### Trend in excess burden of SPC

**Figure 3:**
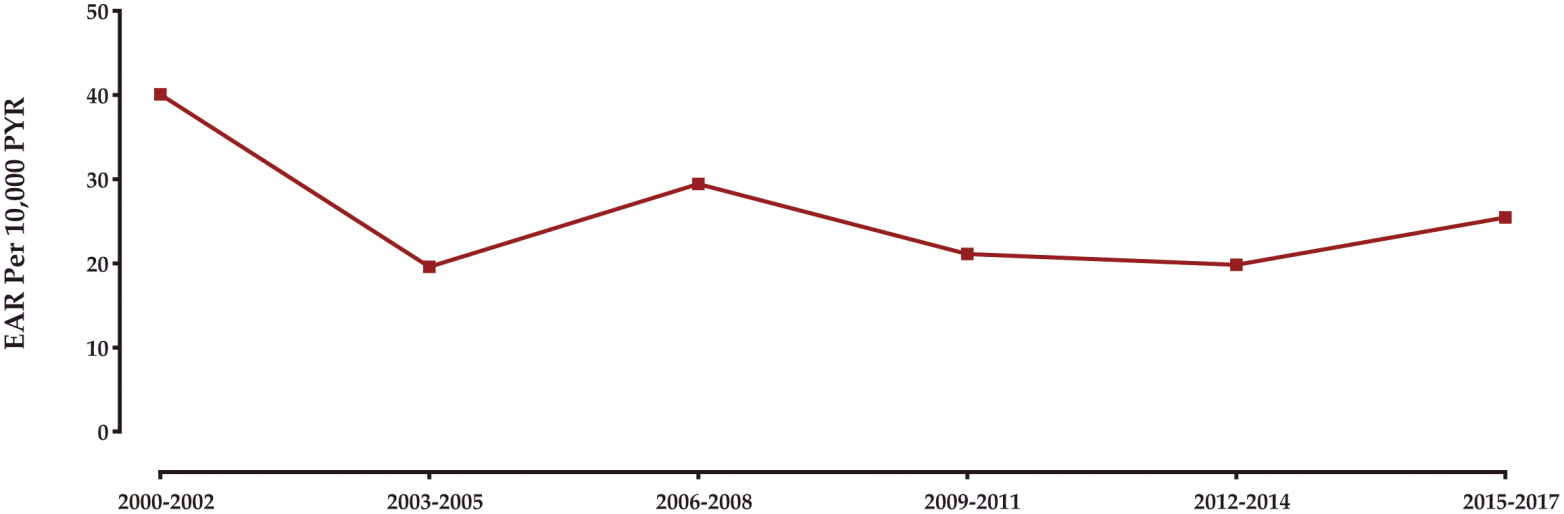
Trend in excess burden of SPCs among rectal NENs survivors.

**Figure 3** illustrates the temporal pattern of the burden of SPCs among rectal NENs survivors in the United States from 2000 to 2018. The analysis reveals that the highest excess burden of SPCs was observed in the years 2000 to 2002. Subsequently, there was a gradual decrease in the burden until 2003-2005. From 2005 onwards, the excess burden of SPCs remained relatively stable, with minor fluctuations.

### Risk of SPC by time latency

**Figure 4:**
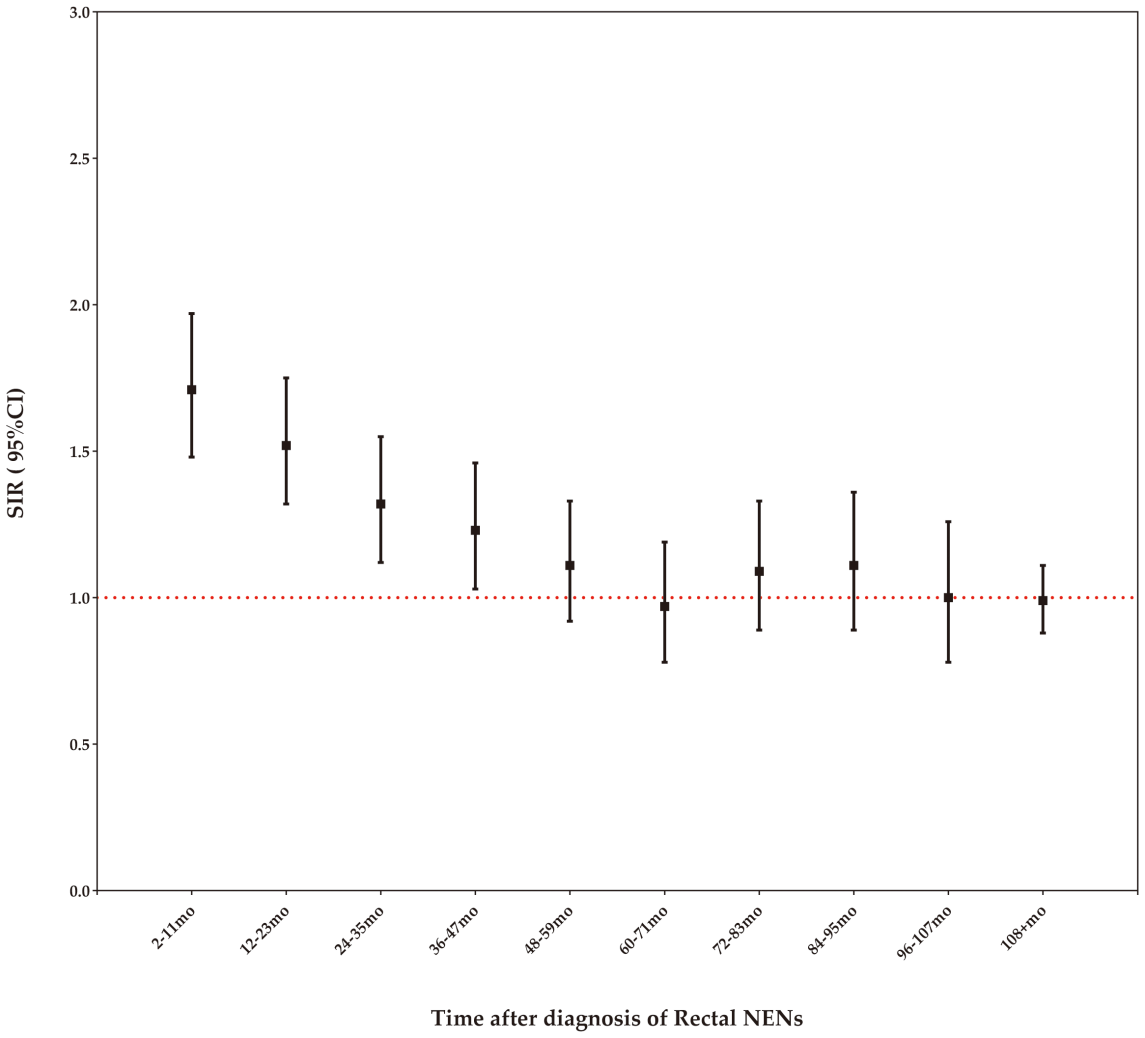
Risk of developing SPCs by time latency after rectal NENs diagnosis.

Our study demonstrates that the risk of SPCs remains elevated compared to the age-matched general US population within the first four years following a rectal NENs diagnosis, but this increased risk diminishes after four years (**Figure 4**).

### Risk of SPC by age at rectal NENs diagnosis

**Figure 5:**
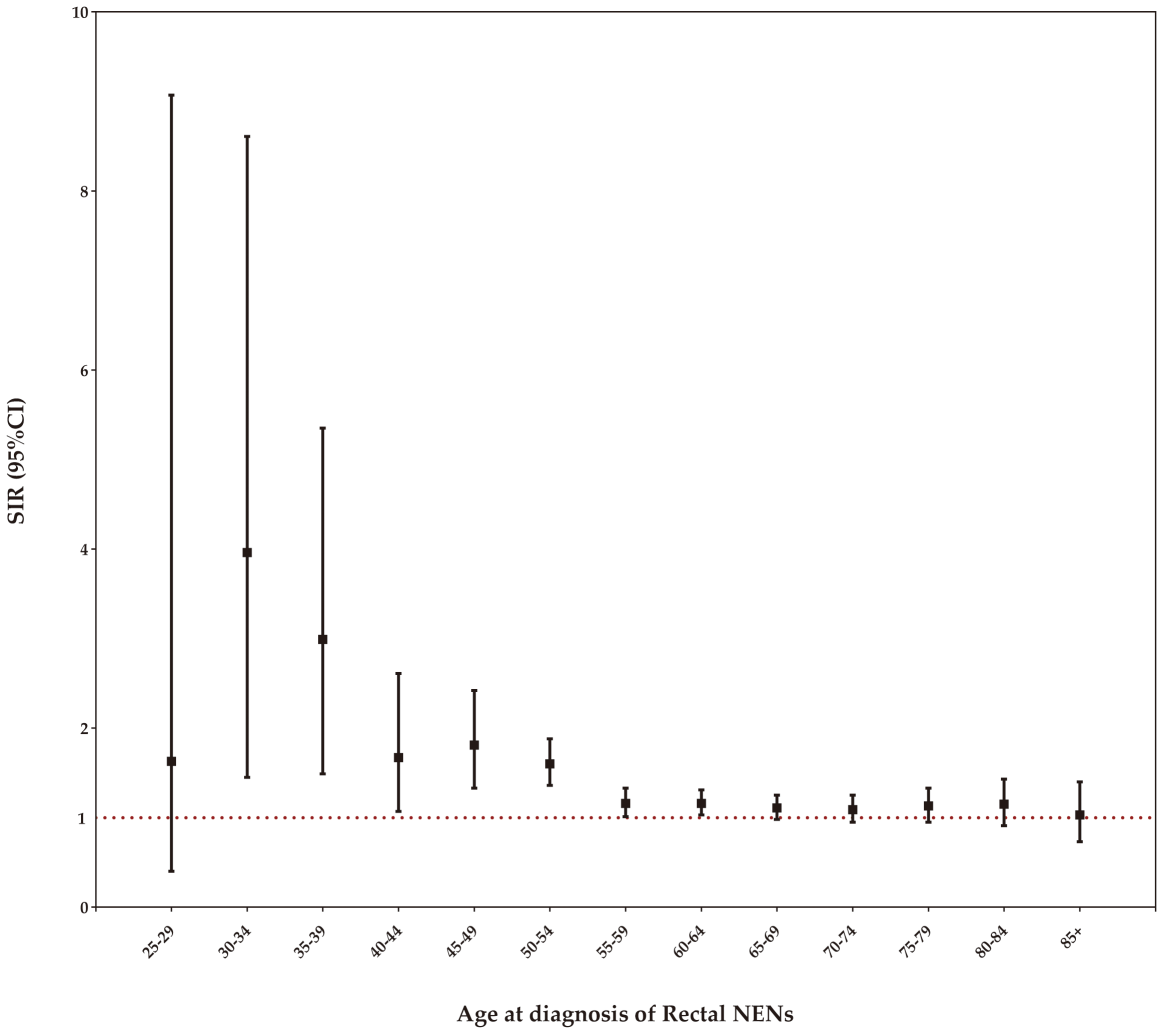
Risk of developing SPCs by age at rectal NENs diagnosis.

Stratifying the SIRs by age at diagnosis of rectal NENs, we observed a statistically significant increase in the incidence of SPCs among patients aged 30-64 years. As age increased within this range, the SIR gradually declined, while still maintaining statistical significance, as demonstrated in **Figure 5**.

## Discussion

As a result of advancements in cancer detection and management, the extended survivorship of cancer patients may have led to an increased risk of developing SPCs (10, 11). A better understanding of the relative risk in this population is crucial for improving lifelong surveillance, especially in diseases where therapeutic innovations have significantly improved the survival from the first primary cancer. This is true for rectal NENs as well, where recent advances in treatments have shown promising results in improving patient outcomes for rectal NENs, including those with high-risk disease (12–15). The occurrence of SPCs may be influenced by shared etiological factors, environmental exposures, and prior cancer treatments. In this study, we assessed the risks of SPCs among patients with a history of rectal NENs in the United States. Our findings revealed an increased relative risk of SPCs among rectal NENs patients (SIR: 1.19, 95%CI: 1.13-1.26, EAR: 21.51 cases per 10000 person-years). This study demonstrates a significant and persistent risk of SPCs among rectal NENs survivors, particularly within the first four years after their cancer diagnosis. These results contribute to the growing body of evidence highlighting the elevated risk of SPCs in cancer survivors. To the best of our knowledge, it is the first study to investigate the relative risk of second cancer development in survivors of rectal NENs based on a large cohort of study participants in the United States.

Our study addresses the question of how to effectively identify rectal NENs patients at a higher risk of developing SPCs. Through analyses of SIR by patient characteristics, we identified significant associations between SPCs and a prior diagnosis of rectal NENs. Male survivors were found to have a higher risk of SPCs diagnosis compared to their female counterparts (SIR: 1.25 versus 1.13). Notably, female patients had a significantly increased risk of developing second thyroid cancer relative to the general US population. Our results also showed that the age at diagnosis of first rectal NENs was a significant factor in the risk of developing SPCs among this population. For individuals aged less than 50 years old, the relative risk of SPCs was significantly elevated by 49% for all cancers combined, compared to matched peers in the general US population. Specially, survivors under the age of 50 at initial cancer diagnosis had a significantly increased risk of subsequent colorectal and pancreatic cancers, but a decreased risk of female genital system cancer. On the other hand, individuals older than 70 years had significantly higher risks of subsequent kidney and renal pelvis cancer, as well as lymphoma, compared to the general population. As the number of younger cancer patients surviving for several decades increases, it becomes crucial to understand the impact of specific SPCs diagnoses on survival, which is essential for tailoring age-specific prevention, screening, and treatment strategies. However, the underlying reasons for this heightened risk still remain unclear, and further studies are needed to better comprehend the diverse age-related risks associated with SPCs.

When developing screening strategies for rectal NENs survivors, it is crucial to consider the time gap between the initial cancer diagnosis and SPCs. Our findings indicate that the risk of SPCs remains elevated compared to the age-matched general population within the first four years following a rectal NENs diagnosis, but this increased risk diminishes after four years. This suggests that the higher incidence of SPCs may be influenced by individual genetic predisposition (16–19). Therefore, implementing focused surveillance during the initial four years is important for promptly detecting potential subsequent cancers in this cohort.

Our study has several limitations that need to be considered when interpreting the results. Firstly, we lacked detailed information on genetic or environmental risk factors and treatment, which may influence the outcomes. Additionally, a 2-month latency period between the initial cancer diagnosis and the identification of subsequent malignancies may have resulted in an overestimation of the true incidence of SPCs. Despite these limitations, our study stands out by examining the association between a first diagnosis of rectal NENs and subsequent malignancies using the largest and most recent cohort from the United States. Furthermore, we provided risk stratification based on patient demographics and characteristics, which enhances awareness regarding SPCs in this specific patient population.

In conclusion, rectal NENs survivors were found to have an increased risk of SPCs compared with the age-matched general US population, especially within the first four years following their initial cancer diagnosis. Furthermore, our study expands upon existing data by demonstrating long-term trends in SPC incidence among rectal NENs survivors, based on a larger patient sample. These findings have the potential to inform further studies on the etiology of SPCs and aid in shaping improved survivorship strategies for patients with rectal NENs. With advancements in cancer treatment and improved survival rates, it is crucial for healthcare practitioners and cancer survivors to be aware of the heightened risk of subsequent primary malignancies. Our findings provide valuable insights that can guide healthcare providers in customizing survivorship care plans to meet individual patient needs and address associated risks. Given the complexities of cancer management, there is a pressing need for research focused on identifying the most appropriate and feasible strategies.

## Data availability statement

Publicly available datasets were analyzed in this study. This data can be found here: SEER programs.

## Author contributions

XZ contributed to the conception. MW performed statistical analysis and wrote the manuscript. JW and ZJ collected the data. WG edited the manuscript. All authors contributed to the article and approved the submitted version.

## References

[1] La Rosa S Uccella S. Classification of neuroendocrine neoplasms: lights and shadows. Rev Endoc Metab Disord (2021) 22(3):527–38. doi: 10.1007/s11154-020-09612-2 PMC8346451 33169199

[2] Rizen EN Phan AT. Neuroendocrine tumors: a relevant clinical update. Curr Oncol Rep (2022) 24(6):703–14. doi: 10.1007/s11912-022-01217-z 35254612

[3] Dasari A Shen C Halperin D Zhao B Zhou S Xu Y. Trends in the incidence, prevalence, and survival outcomes in patients with neuroendocrine tumors in the United States. JAMA Oncol (2017) 3(10):1335–42. doi: 10.1001/jamaoncol.2017.0589 PMC5824320 28448665

[4] Yao JC Hassan M Phan A Dagohoy C Leary C Mares JE. One hundred years after “carcinoid”: epidemiology of and prognostic factors for neuroendocrine tumors in 35,825 cases in the United States. J Clin Oncol (2008) 26(18):3063–72. doi: 10.1200/JCO.2007.15.4377 18565894

[5] Das S Dasari A. Epidemiology, incidence, and prevalence of neuroendocrine neoplasms: are there global differences? Curr Oncol Rep (2021) 23(4):43. doi: 10.1007/s11912-021-01029-7 33719003 PMC8118193

[6] Lee MR Harris C Baeg KJ Aronson A Wisnivesky JP Kim MK. Incidence trends of gastroenteropancreatic neuroendocrine tumors in the United States. Clin Gastroenterol Hepatol (2019) 17(11):2212–2217.e2211. doi: 10.1016/j.cgh.2018.12.017 30580091

[7] Xu Z Wang L Dai S Chen M Li F Sun J. Epidemiologic trends of and factors associated with overall survival for patients with gastroenteropancreatic neuroendocrine tumors in the United States. JAMA network Open (2021) 4(9):e2124750. doi: 10.1001/jamanetworkopen.2021.24750 34554237 PMC8461504

[8] Clift AK Drymousis P Al-Nahhas A Wasan H Martin J Holm S. Incidence of second primary Malignancies in patients with neuroendocrine tumours. Neuroendocrinology (2015) 102(1-2):26–32. doi: 10.1159/000381716 25824138

[9] Kamp K Damhuis RA Feelders RA de Herder WW. Occurrence of second primary Malignancies in patients with neuroendocrine tumors of the digestive tract and pancreas. Endocrine-related Canc (2012) 19(1):95–9. doi: 10.1530/ERC-11-0315 22194442

[10] Sung H Hyun N Leach CR Yabroff KR Jemal A. Association of first primary cancer with risk of subsequent primary cancer among survivors of adult-onset cancers in the United States. Jama (2020) 324(24):2521–35. doi: 10.1001/jama.2020.23130 PMC7756242 33351041

[11] Sung H Siegel RL Hyun N Miller KD Yabroff KR Jemal A. Subsequent primary cancer risk among 5-year survivors of adolescent and young adult cancers. J Natl Cancer Institute. (2022) 114(8):1095–108. doi: 10.1093/jnci/djac091 PMC9360462 35511931

[12] Gallo C Rossi RE Cavalcoli F Barbaro F Boškoski I Invernizzi P. Rectal neuroendocrine tumors: Current advances in management, treatment, and surveillance. World J Gastroenterol (2022) 28(11):1123–38. doi: 10.3748/wjg.v28.i11.1123 PMC8985485 35431507

[13] Maione F Chini A Milone M Gennarelli N Manigrasso M Maione R. Diagnosis and management of rectal neuroendocrine tumors (NETs). Diagnost (Basel Switzerland). (2021) 11(5):771. doi: 10.3390/diagnostics11050771 PMC8145857 33923121

[14] Basuroy R Haji A Ramage JK Quaglia A Srirajaskanthan R. Review article: the investigation and management of rectal neuroendocrine tumours. Alimentary Pharmacol Ther (2016) 44(4):332–45. doi: 10.1111/apt.13697 27302838

[15] Bertani E Ravizza D Milione M Massironi S Grana CM Zerini D. Neuroendocrine neoplasms of rectum: A management update. Cancer Treat Rev (2018) 66:45–55. doi: 10.1016/j.ctrv.2018.04.003 29684743

[16] Travis LB Rabkin CS Brown LM Allan JM Alter BP Ambrosone CB. Cancer survivorship–genetic susceptibility and second primary cancers: research strategies and recommendations. J Natl Cancer Institute. (2006) 98(1):15–25. doi: 10.1093/jnci/djj001 16391368

[17] Travis LB Demark Wahnefried W Allan JM Wood ME Ng AK. Aetiology, genetics and prevention of secondary neoplasms in adult cancer survivors. Nat Rev Clin Oncol (2013) 10(5):289–301. doi: 10.1038/nrclinonc.2013.41 23529000

[18] Oeffinger KC Baxi SS Novetsky Friedman D Moskowitz CS. Solid tumor second primary neoplasms: who is at risk, what can we do? Semin Oncol (2013) 40(6):676–89. doi: 10.1053/j.seminoncol.2013.09.012 PMC3921623 24331190

[19] Wang Z Wilson CL Easton J Thrasher A Mulder H Liu Q. Genetic risk for subsequent neoplasms among long-term survivors of childhood cancer. J Clin Oncol (2018) 36(20):2078–87. doi: 10.1200/JCO.2018.77.8589 PMC6036620 29847298

